# Relevance of histopathological findings for predictive scoring of short-term treatment response to plasma exchange in severe ANCA-associated renal vasculitides

**DOI:** 10.3389/fimmu.2024.1340999

**Published:** 2024-02-06

**Authors:** Samy Hakroush, Peter Korsten, Philipp Ströbel, Björn Tampe

**Affiliations:** ^1^ Institute of Pathology, University Medical Center Göttingen, Göttingen, Germany; ^2^ SYNLAB Pathology Hannover, SYNLAB Holding Germany, Augsburg, Germany; ^3^ Institute of Pathology, Klinikum Bremen-Mitte, School of Medicine of the University of Göttingen, Bremen, Germany; ^4^ Department of Nephrology and Rheumatology, University Medical Center Göttingen, Göttingen, Germany; ^5^ Department of Rheumatology and Clinical Immunology, St Josef-Stift Sendenhorst, Sendenhorst, Germany

**Keywords:** ANCA-associated renal vasculitis, AAV, rapid-progressive glomerulonephritis, RPGN, therapeutic plasma exchange, plex, clinicopathological scoring

## Abstract

**Introduction:**

Rapidly progressive glomerulonephritis (RPGN) is characterized by a rapid loss of kidney function, affecting both renal and overall patient survival. Antineutrophil cytoplasmic antibody (ANCA)-associated vasculitis (AAV) is a small vessel vasculitis affecting multiple organ systems including the kidney, and among most frequent causes of RPGN. We here aimed to validate a recently described scoring system for short-term treatment response to therapeutic plasma exchange (PLEX) in a well-characterized and independent cohort of severe renal AAV presenting with RPGN. Furthermore, we compared this scoring with established classification systems in renal AAV including histopathological findings.

**Methods:**

We here directly compare the scoring system with retrospective data about PLEX treatment in our own clinical practice and according to current recommendations in a cohort of 53 patients with severe AAV presenting with RPGN confirmed by kidney biopsy.

**Results:**

We here confirm that PLEX scoring is capable to identify patients at risk for short-term poor outcome in severe AAV presenting with RPGN (*p<0.0001*). Furthermore, multiple stepwise regression analysis revealed that the PLEX score with renal biopsy performed best to predict poor outcome in this patient population (*p<0.0001*).

**Conclusion:**

Our observations underscore the relevance of performing a kidney biopsy in this patient population that is often challenged in the setting of intensive care treatment, requirement of KRT with need for anticoagulation and bleeding risk. Therefore, validation of our observations and this recent scoring system for treatment response to PLEX in independent cohorts would be of great clinical relevance in the treatment of patients with severe AAV presenting with RPGN.

## Introduction

Rapidly progressive glomerulonephritis (RPGN) is an acute kidney injury (AKI) condition that significantly impacts both, renal and overall patient survival. A primary contributor to RPGN is antineutrophil cytoplasmic antibody (ANCA)-associated vasculitis (AAV), a small vessel vasculitis affecting various organ systems, prominently the kidneys. Berden et al. classified renal involvement in AAV based on histopathological features into four classes (focal, crescentic, mixed, and sclerotic), with the sclerotic class (more than 50% sclerotic glomeruli) associated with the poorest long-term renal survival rates (1).

To enhance predictive accuracy for end-stage kidney disease (ESKD) in AAV patients, Brix et al. proposed the ANCA renal risk score (ARRS), incorporating baseline glomerular filtration rate (GFR) alongside histopathological findings such as the percentage of normal glomeruli and tubular atrophy/interstitial fibrosis (2). While these classifications are geared towards predicting long-term ESKD, a subgroup of severe AAV presents with RPGN necessitating kidney replacement therapy (KRT) during the initial disease phase (3). Given the impact of disease severity on AKI, KRT requirement, and short-term renal recovery in critically ill patients, identifying predictors for KRT requirement and renal recovery after initiating remission induction therapy is crucial (4). Recommended regimens for severe AAV involve aggressive immunosuppressive therapy to improve outcomes (5). However, some patients may still require KRT despite intensive immunosuppressive therapy. In such cases, therapeutic plasma exchange (PLEX) is recommended to deplete pathogenic ANCA autoantibodies, particularly in instances of severe deterioration of kidney function due to RPGN in new onset or relapsing disease (5–9). The MEPEX trial demonstrated that PLEX increased renal recovery rates in severe AAV cases though long-term outcomes (death or ESKD) did not significantly differ among treatment groups (9, 10). These findings were recently reinforced by the PEXIVAS trial, which reported no long-term benefits in outcomes for patients receiving PLEX in addition to standard immunosuppressive therapy (11). However, the inclusion of patients with less severe renal dysfunction may limit the generalizability of these findings to critically ill patients at risk for KRT and death. Nevertheless, PEXIVAS appears to confirm data from MEPEX, suggesting that PLEX can temporarily reduce the risk of ESKD and supported by a meta-analysis of seven trials with 999 participants indicating a reduced risk of ESKD at 12 months with PLEX (11, 12). In this context, an easily applicable scoring system has been proposed to identify patients who would benefit from PLEX (13). Using a model dependent on covariables, the average treatment effect of PLEX for those with recommended treatment showed an absolute risk reduction for KRT or death after 12 months by 24.6% (13). This study aims to validate these findings regarding the short-term treatment response to PLEX in a well-characterized and independent cohort of severe renal AAV presenting with RPGN (14, 15). Additionally, we compare this scoring system with established classification systems in renal AAV, including histopathological findings.

## Methods

### Study population

We here directly compare the scoring system with retrospective data about PLEX treatment in our own clinical practice and according to current recommendations in patients presenting with a serum creatinine levels above 5.7 mg/dL (500 μmol/L) and/or requirement of KRT in a cohort of 53 AAV patients with RPGN confirmed by kidney biopsy, detailed information about critical illness in this patient cohort has recently been described (14–16).

### Histopathological examination

Two renal pathologists independently assessed kidney biopsies while being unaware of the data analysis. Each glomerulus within a renal biopsy specimen was individually evaluated for the presence of necrosis, crescents, and global sclerosis. Subsequently, the percentage of glomeruli exhibiting any of these features was determined as a fraction of the total number of glomeruli in each renal biopsy. In addition to these categories, the extent of interstitial fibrosis/tubular atrophy (IF/TA) was quantified. The histopathological subgrouping, following the criteria of Berden et al. (focal, crescentic, mixed, or sclerotic class), and the ARRS classification as per Brix et al. (low, medium, or high risk), were then performed based on these assessments (1, 2). As described recently, scoring to predict treatment response to PLEX was performed accordingly (individual items are presented in **Tables 1**, **2**) (13).

**Table 1:** Variables for the score without renal biopsy.

Variable	Total cohort n=53	PLEX-R n=11	No PLEX-R n=42
Age >45 years − n (%) MPA vasculitis − n (%) PR3-ANCA positive − n (%) MPO-ANCA positive − n (%) Serum creatinine 251-400 µmol/L − n (%) Serum creatinine 401-600 µmol/L − n (%) Serum creatinine >600 µmol/L − n (%)	47 (88.7) 26 (49.1) 27 (50.9) 26 (49.1) 13 (24.5) 9 (17) 6 (11.3)	10 (90.9) 10 (90.9) 0 (0) 11 (100) 0 (0) 5 (45.5) 6 (54.5)	37 (88.1) 16 (38.1) 27 (64.3) 15 (35.7) 13 (31) 4 (9.5) 0 (0)

ANCA, antineutrophil cytoplasmic antibody; MPA, microscopic polyangiitis; MPO, myeloperoxidase; PR3, proteinase 3.

**Table 2:** Variables for the score with renal biopsy.

Variable	Total cohort n=53	PLEX-R n=24	No PLEX-R n=29
Male sex − n (%) MPA vasculitis − n (%) RLV − n (%) PR3-ANCA positive − n (%) MPO-ANCA positive − n (%) Serum creatinine 251-400 µmol/L − n (%) Serum creatinine 401-600 µmol/L − n (%) Serum creatinine >600 µmol/L − n (%) Brix score ≥7 − n (%) Berden classification: crescentic class − n (%) Berden classification: mixed class − n (%)	30 (56.6) 26 (49.1) 9 (17) 27 (50.9) 26 (49.1) 13 (24.5) 9 (17) 6 (11.3) 10 (18.9) 17 (32.1) 7 (13.2)	18 (75) 17 (70.8) 8 (33.3) 8 (33.3) 16 (66.7) 8 (33.3) 9 (37.5) 6 (25) 10 (41.7) 16 (66.7) 1 (4.2)	12 (41.4) 9 (31) 1 (3.4) 19 (65.5) 10 (34.5) 5 (17.2) 0 (0) 0 (0) 0 (0) 1 (3.4) 6 (20.7)

ANCA, antineutrophil cytoplasmic antibody; MPA, microscopic polyangiitis; MPO, myeloperoxidase; PR3, proteinase 3:; RLV, renal limited vasculitis.

### ANCA autoantibody measurements

MPO-ANCA (reference range, <3.5 IU/mL) and PR3-ANCA autoantibodies (reference range, <2 IU/mL) were measured by immunoassay (ImmunoCAP 250, Thermo Fisher Scientific, Waltham, MA, USA).

### Statistical methods

Variables were tested for normal distribution using the Shapiro–Wilk test, statistical comparisons were not formally powered or prespecified. Survival-curve analyses were performed using the Kaplan-Meier method, comparison of survival curves was performed with log rank (Mantel-Cox) testing. Data analyses were performed with GraphPad Prism (version 8.4.3 for MacOS, GraphPad Software, San Diego, California, USA). Multiple regression analyses were performed using IBM SPSS Statistics (version 27 for MacOS, IBM Corporation, Armonk, NY, USA). A probability (*p*) value of <0.05 was considered statistically significant.

## Results

### PLEX scoring identifies patients at risk for poor short-term outcome in severe AAV

**Figure 1:**
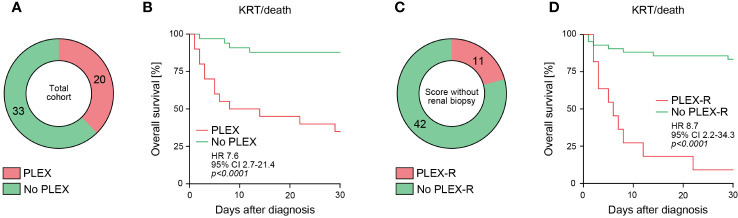
PLEX scoring identifies patients at risk for poor short-term outcome in severe AAV. **(A)** Among the total number of 53 patients with severe AAV presenting with RPGN, subgroups receiving PLEX or not are shown. **(B)** Overall survival (KRT or death) within 30 days after diagnosis according to PLEX treatment or not are shown, comparison of survival curves was performed with log rank (Mantel-Cox) testing. **(C)** Among the total number of 53 patients with severe AAV presenting with RPGN, subgroups where PLEX was recommended (PLEX-R) or not are shown. **(D)** Overall survival (KRT or death) within 30 days after diagnosis according to PLEX recommendation (PLEX-R) or not are shown, comparison of survival curves was performed with log rank (Mantel-Cox) testing.

In the total cohort, 20/53 (37.7%) of AAV patients received PLEX treatment (**Figure 1A**). Because PLEX in AAV is recommended in severe organ failure, short-term outcome within 30 days after diagnosis revealed that the outcome was worse in the PLEX-treated subgroup (requirement of KRT or death, *p<0.0001*, **Figure 1B**). Group separation according to recently described scoring of patients that could benefit from PLEX (PLEX-R) confirmed that short-term outcome was poor in the PLEX-R subgroup as compared to patients where PLEX was not recommended (no PLEX-R, *p<0.0001*, **Table 1**, **Figures 1C, D**). These observations confirmed that PLEX scoring is capable to identify patients at risk for short-term poor outcome in severe AAV presenting with RPGN.

### Histopathological scoring of RPGN in severe AAV predicts short-term outcome in severe AAV

**Figure 2:**
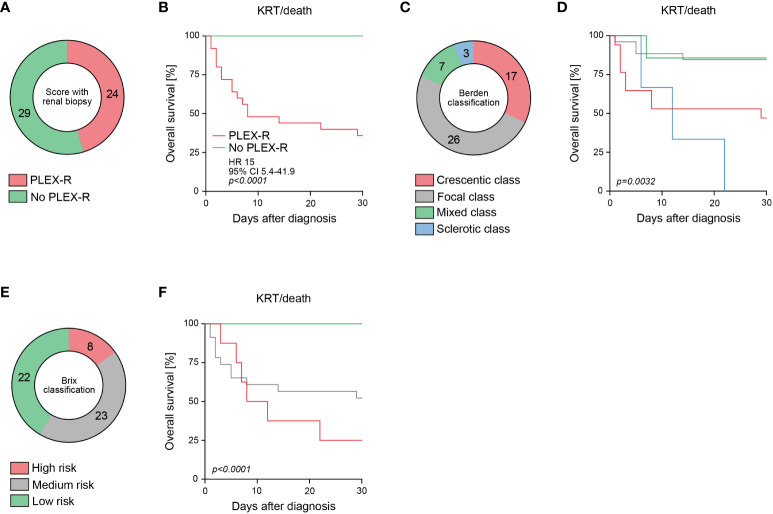
Histopathological scoring of RPGN in severe AAV predicts short-term outcome in severe AAV. **(A)** Among the total number of 53 patients with severe AAV presenting with RPGN, subgroups where PLEX was recommended (PLEX-R) according to scoring with renal biopsy are shown. **(B)** Overall survival (KRT or death) within 30 days after diagnosis according to PLEX recommendation (PLEX-R) including histopathology are shown, comparison of survival curves was performed with log rank (Mantel-Cox) testing. **(C, D)** Classification according to Berden and overall survival (KRT or death) within 30 days after diagnosis are shown, comparison of survival curves was performed with log rank (Mantel-Cox) testing. **(E, F)** Classification according to Brix and overall survival (KRT or death) within 30 days after diagnosis are shown, comparison of survival curves was performed with log rank (Mantel-Cox) testing.

Next, we analyzed the PLEX score with renal biopsy (13). By including histopathological scoring, PLEX was recommended in 24/53 (45.3%) of patients (PLEX-R, **Table 2**, **Figure 2A**). Again, we observed a strong association with short-term outcome by including histopathological classifications (*p<0.0001*, **Figure 2B**). Direct assessment of the Berden classification revealed poorest outcomes in crescentic and sclerotic class RPGN (*p=0.0032*, **Figures 2C, D**) (1). In addition, the Brix classification was capable to predict short-term outcome in severe AAV (*p<0.0001*, **Figures 2E, F**) (2). In summary, these observations indicate that PLEX scoring with renal biopsy and the Brix classification effectively identified patients at risk for poor short-term outcome in severe RPGN.

### PLEX score with renal biopsy is superior for short-term outcome prediction as compared to Berden and Brix classifications in severe AAV

Next, we aimed to identify the best prediction of short-term outcome by comparing all these scoring systems. As assessed by multiple regression, the PLEX score with renal biopsy was superior to identify patients at risk for KRT or death in severe AAV (*p<0.0001*) as compared to PLEX scoring without renal biopsy (*p=0.3246*), or classification according to Berden (crescentic class: *p=0.2453*, sclerotic class: *p=0.7212*) and Brix (high risk class: *p=0.9621*, **Table 3**) (1, 2). In summary, we here validate application of the PLEX score for outcome prediction in severe AAV presenting with RPGN. Furthermore, comparative analysis revealed that the PLEX score with renal biopsy performed best to predict poor outcome in this patient population.

**Table 3:** Multiple comparison between KRT/death and scoring systems for renal AAV.

	ß	p value
PLEX-R without renal biopsy PLEX-R with renal biopsy Berden classification: crescentic class Berden classification: sclerotic class Brix classification: high risk	0.1095 0.7826 -0.2001 -0.0540 -0.0070	*0.3246* *<0.0001* *0.2453* *0.7212* *0.9621*

AAV, ANCA-associated vasculitis; PLEX, therapeutic plasma exchange; PLEX-R, PLEX recommended.

### Comparative analysis of recommendation and real-life performance of PLEX in severe AAV with RPGN

**Figure 3:**
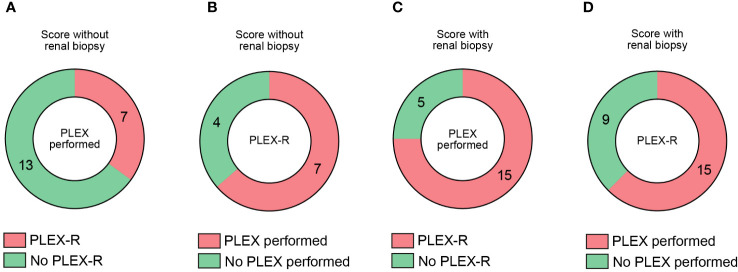
Comparative analysis of recommendation and real-life performance of PLEX in severe AAV with RPGN. **(A)** Among 20 patients receiving PLEX, subgroups where PLEX was recommended (PLEX-R) or not are shown. **(B)** Among 11 patients where PLEX was recommended (PLEX-R), subgroups where PLEX was performed or not are shown. **(C)** Among 20 patients receiving PLEX, subgroups where PLEX was recommended (PLEX-R) according to scoring with renal biopsy are shown. **(D)** Among 24 patients where PLEX was recommended (PLEX-R) according to scoring with renal biopsy are shown, subgroups where PLEX was performed or not are shown.

Based on our observations that PLEX scoring effectively identified patients at risk for poor short-term outcome in severe AAV presenting with RPGN, we finally compared recommendation of PLEX according to these scorings and real-time performance of PLEX in this retrospective patient cohort (13). PLEX scoring without renal biopsy revealed that PLEX was recommended (PLEX-R) in 7/20 (35%), while it was not recommended in 13/20 (65%) of patients that received PLEX (**Figure 3A**). Among the PLEX-R subgroup, PLEX was performed in 7/11 (63.6%, **Figure 3B**). When PLEX was performed, PLEX was recommended in 15/20 (75%) of patients (PLEX-R, **Figure 3C**). In the PLEX-R subgroup, PLEX was performed in 15/24 (62.5%) of patients (**Figure 3D**). These observations suggest that PLEX was performed in a considerable number of cases with severe AAV presenting with RPGN although not recommended according to predictive scoring of treatment response.

## Discussion

For many years, PLEX has been primarily administered based on the extent of kidney involvement, with recommendations left to the discretion of treating physicians in patients with severe AAV presenting with RPGN (14). RPGN is a common manifestation in severe AAV and is linked to heightened morbidity and mortality. Kidney biopsy is frequently conducted in AAV cases to confirm the diagnosis of pauci-immune and crescentic RPGN. Beyond its diagnostic value, kidney biopsy also furnishes dependable prognostic information for predicting renal outcomes, as validated by the Berden and Brix classifications (1, 2).

The rationale behind employing PLEX in AAV is robust, particularly given evidence demonstrating the pathogenic role of ANCA autoantibodies in animal models (17). Therefore, there is a hypothesis that the early initiation of PLEX, aimed at removing ANCA autoantibodies, could enhance patient outcomes, especially during the period when concurrent immunosuppression is expected to be effective. Recent reports indicate that PLEX treatment did not yield an overall superior outcome in AAV patients, consistent with findings from the PEXIVAS trial (11, 13). Nevertheless, PLEX demonstrated a tendency to be linked with lower incidences of KRT or death, although statistical significance was not achieved (11). Intriguingly, a subset of patients with aggressive kidney disease and minimal scarring seemed to benefit from PLEX. These patients were effectively identified using a scoring system that integrates baseline characteristics and renal histopathologic findings (13). Notably, in the sclerotic class, the addition of histopathological findings indicated that PLEX did not correlate with improved outcomes (13). Conversely, when considering the entire AAV population, PLEX treatment did not show a superior outcome at 12 months concerning KRT or death (13). These results align with the earlier mentioned PEXIVAS trial and underscore the significance of kidney biopsy findings in predicting short-term treatment response to PLEX in renal AAV (11, 13).

By application of this PLEX scoring system, we here confirmed that PLEX scoring is capable to identify patients at short-term risk for KRT or death in severe AAV presenting with RPGN. Furthermore, the PLEX scoring identified a considerable subset of patients that could benefit from PLEX (although not treated), or not benefit from PLEX (although treated). This observation is especially relevant for treatment choice in severe AAV since PLEX may also cause severe side effects including increased risk for serious infections (12). Finally, we here show that the PLEX scoring that includes histopathological data is superior to predict KRT or death in patients with severe AAV presenting with RPGN as compared to PLEX scoring without renal biopsy, or classification according to Berden and Brix. This underscores the relevance of performing a kidney biopsy in this patient population that is often challenged in the setting of intensive care treatment, requirement of KRT with need for anticoagulation and bleeding risk. We are aware that these conclusions are derived from a relatively small patient cohort. However, we here particularly included patients with severe AAV presenting with RPGN where PLEX was performed in a considerable number of cases. Therefore, validation of our observations and this recent scoring system for treatment response to PLEX in independent cohorts would be of great clinical relevance in the treatment of patients with severe AAV presenting with RPGN.

## Data availability statement

The original contributions presented in the study are included in the article/supplementary material. Further inquiries can be directed to the corresponding author.

## Ethics statement

The studies involving humans were approved by the Institutional Review Board of the University Medical Center Göttingen, Germany. The studies were conducted in accordance with the local legislation and institutional requirements. The participants provided their written informed consent to participate in this study.

## Author contributions

SH: Data curation, Writing – review & editing. PK: Writing – review & editing. PS: Data curation, Writing – review & editing. BT: Conceptualization, Data curation, Formal Analysis, Funding acquisition, Investigation, Methodology, Project administration, Resources, Software, Supervision, Validation, Visualization, Writing – original draft.
